# Japanese Society of Medical Oncology/Japan Society of Clinical Oncology/Japanese Society of Pediatric Hematology/Oncology-led clinical recommendations on the diagnosis and use of tropomyosin receptor kinase inhibitors in adult and pediatric patients with neurotrophic receptor tyrosine kinase fusion-positive advanced solid tumors

**DOI:** 10.1007/s10147-023-02345-7

**Published:** 2023-05-22

**Authors:** Yoichi Naito, Saori Mishima, Kiwamu Akagi, Naomi Hayashi, Akira Hirasawa, Tomoro Hishiki, Ataru Igarashi, Masafumi Ikeda, Shigenori Kadowaki, Hiroaki Kajiyama, Motohiro Kato, Hirotsugu Kenmotsu, Yasuhiro Kodera, Keigo Komine, Takafumi Koyama, Osamu Maeda, Mitsuru Miyachi, Hiroshi Nishihara, Hiroyuki Nishiyama, Shouichi Ohga, Wataru Okamoto, Eiji Oki, Shigeru Ono, Masashi Sanada, Ikuo Sekine, Tadao Takano, Kayoko Tao, Keita Terashima, Katsuya Tsuchihara, Yasushi Yatabe, Takayuki Yoshino, Eishi Baba

**Affiliations:** 1 National Cancer Center Hospital East, Kashiwa, Japan; 2 Saitama Cancer Center, Saitama, Japan; 3 The Cancer Institute Hospital of Japanese Foundation for Cancer Research, Tokyo, Japan; 4 Okayama University, Okayama, Japan; 5 Chiba University, Chiba, Japan; 6 Yokohama City University School of Medicine, Yokohama, Japan; 7 Aichi Cancer Center, Aichi, Japan; 8 Nagoya University, Aichi, Japan; 9 The University of Tokyo, Tokyo, Japan; 10 Shizuoka Cancer Center, Shizuoka, Japan; 11 Nagoya University Hospital, Aichi, Japan; 12 Tohoku University, Miyagi, Japan; 13 National Cancer Center Hospital, Tokyo, Japan; 14 Kyoto Prefectural University of Medicine, Kyoto, Japan; 15 Keio University, Tokyo, Japan; 16 Tsukuba University, Ibaraki, Japan; 17 Kyushu University, Fukuoka, Japan; 18 Hiroshima University Hospital, Hiroshima, Japan; 19 Jichi Medical University, Tochigi, Japan; 20 National Hospital Organization Nagoya Medical Center, Aichi, Japan; 21 National Center for Child Health and Development, Tokyo, Japan; 22 Department of Oncology and Social Medicine, Graduate School of Medical Sciences, Kyushu University, Fukuoka, Japan

**Keywords:** Advanced solid tumor, Clinical practice guideline, Neurotrophic receptor tyrosine kinase (NTRK) fusion, Tropomyosin receptor kinase (TRK) inhibitor, Tumor-agnostic treatment

## Abstract

**Background:**

Clinical trials have reported the efficacy of tropomyosin receptor kinase (TRK) inhibitors against neurotrophic receptor tyrosine kinase (NTRK) fusion gene-positive advanced solid tumors. The accumulated evidence of tumor-agnostic agent has made since TRK inhibitors were approved and used in clinical practice. Therefore, we have revised the ‘Japan Society of Clinical Oncology (JSCO)/Japanese Society of Medical Oncology (JSMO)-led clinical recommendations on the diagnosis and use of tropomyosin receptor kinase inhibitors in adult and pediatric patients with neurotrophic receptor tyrosine kinase fusion-positive advanced solid tumors, cooperated by the Japanese Society of Pediatric Hematology/Oncology (JSPHO)’.

**Methods:**

Clinical questions regarding medical care were formulated for patients with NTRK fusion-positive advanced solid tumors. Relevant publications were searched by PubMed and Cochrane Database. Critical publications and conference reports were added manually. Systematic reviews were performed for each clinical question for the purpose of developing clinical recommendations. The committee members identified by JSCO, JSMO, and JSPHO voted to determine the level of each recommendation considering the strength of evidence, expected risks and benefits to patients, and other related factors. Thereafter, a peer review by experts nominated from JSCO, JSMO, and JSPHO, and the public comments among all societies' members was done.

**Results:**

The current guideline describes 3 clinical questions and 14 recommendations for whom, when, and how NTRK fusion should be tested, and what is recommended for patients with NTRK fusion-positive advanced solid tumors.

**Conclusion:**

The committee proposed 14 recommendations for performing NTRK testing properly to select patients who are likely to benefit from TRK inhibitors.

## Introduction

Historically, cancer care has been conducted based on the multifaceted evaluation of a case, such as the pathological diagnosis and staging of the disease, benefits and risks of treatments, and the patient's preference. The identification of the primary site and determination of histological type are important clinical information that forms the basis for determining treatment strategy. A recent advance in molecular biology has revealed the various biological characteristics of tumors and has enabled clinical development of tumor-agnostic drugs beyond the organ specificity of diseases.

The efficacy of tropomyosin receptor kinase (TRK) inhibitors against neurotrophic receptor tyrosine kinase (NTRK) fusion gene-positive advanced solid cancers was demonstrated, and the U.S. Food and Drug Administration (FDA) approved larotrectinib in November 2018 and entrectinib in August 2019. Larotrectinib was also approved by European Medicines Agency (EMA) in September 2019. In Japan, entrectinib was approved in June 2019, which was earliest in the world. Entrectinib was the second tumor-agnostic drug approved in Japan. Moreover, larotrectinib was approved as a second TRK inhibitor for NTRK fusion-positive solid tumors.

The present guidelines provide a guide to diagnosis and treatment and should be utilized in clinical practice according to the recommendation levels described and by adjusting them for individual patients. They are expected to contribute to improving treatment outcomes in patients with solid cancer by utilizing them to perform appropriate tests and treatments on appropriate patients at appropriate timing.

## Materials and methods

The current guidelines systematically describe items to be considered when treating patients with NTRK fusion-positive solid tumors, including the timing and methods of testing *NTRK* fusions, the positioning of immunotherapy. In the clinical setting in Japan, if appropriate tests are performed on appropriate patients and the patients receive appropriate treatment at appropriate timing based on the recommended levels described in the present guidelines, treatment outcomes in patients with solid tumors are expected to be improved.

In the preparation of these guidelines, clinical questions (CQs) were set, and regarding evidence that provides the basis for the answers to those questions, the literature was collected by handsearches and subjected to a systematic review. In setting the CQs, the working group of the Clinical Practice Guidelines for Tumor-Agnostic Genomic Medicine in Adult and Pediatric Patients with Advanced Solid Tumors (3rd edition) prepared draft CQs and decided which ones would be included in the guidelines.

Keywords related to each CQ were selected and sent to the Japan Medical Library Association, which generated queries used to perform comprehensive literature searches. The PubMed, Ichushi Web, and Cochrane Library databases were used in the searches. Important reports by various academic societies were also collected by handsearches and used in the guidelines. Primary and secondary screenings and systematic reviews were performed by persons in charge (SM/YN) of the working group of the Clinical Practice Guidelines for Tumor-Agnostic Genomic Medicine in Adult and Pediatric Patients with Advanced Solid Tumors (3rd edition). The recommendation levels specified for the CQs were determined by voting by the committee members (Table 1). The levels, which were determined based on factors such as the strength of the evidence and the expected benefits and disadvantages for patients, are as follows: strongly recommended (SR), recommended (R), expert consensus opinion (ECO), and not recommended (NR). The status of regulatory approval and insurance coverage in Japan for the treatments (including indications for testing and treatment) was not considered during the voting but was indicated in the remarks section as needed. The overall assessments based on voting were as follows: (1) SR if ≥ 70% of the votes were for SR; (2) R if criterion (1) was not met and SR votes + R votes accounted for ≥ 70% of the total; (3) ECO if criteria (1) and (2) were not met and SR votes + R votes + ECO votes accounted for ≥ 70% of the total; and (4) NR if NR accounted for ≥ 50% of the total regardless of whether criteria (1), (2), or (3) were met. If all of the criteria (1)–(4) were not met, the assessment was “no recommendation level.”

**Table 1:** Degrees of recommendation and decision criteria

Degree of recommendation	Decision criteria
Strongly recommended [SR]	There is sufficient evidence and the benefits of testing outweigh the losses for patients
Recommended [R]	There is certain evidence, considering the balance between benefits and losses for patients
Expert consensus opinion [ECO]	A certain consensus has been obtained although evidence and information that shows patient benefits cannot be said to be sufficient
Not recommended [NR]	There is evidence against efficacy or for adverse outcome, generally not recommended

The recommendations for the CQs include recommendations that are not currently based on strong evidence. As new evidence accumulates, the information and recommendations in these guidelines may change significantly. Although these guidelines will be updated as appropriate, in using a drug clinically, the latest medical information should be reviewed, and every effort made to ensure the drug is used properly.

## Results

### What is *NTRK*?

The *NTRK1* gene was discovered in a gene transfer assay using colorectal cancer tissue and reported as a cancer gene, OncB, by Pulciani, Barbacid, et al. in 1982 [1]. *NTRK* gene family members known to date are *NTRK1–3* (Table 2). *NTRK1–3* encode tyrosine receptor kinases, tropomyosin receptor kinase (TRK) A, TRKB, and TRKC, respectively. TRKA is expressed in the nervous system and gets phosphorylated when nerve growth factor (NGF) binds to it [2, 3]. Known ligands are brain-derived neurotrophic factor (BDNF) and neurotrophin (NT)-4 for TRKB and NT-3 for TRKC. Although NT-3 binds to other TRKs, it has the highest affinity with TRKC. TRKA regulates pain and body temperature, TRKB controls movement, memory, emotion, appetite, and body weight, and TRKC affects proprioception. The binding of a ligand to TRK induces the autophosphorylation of intracellular tyrosine residues, which activates downstream pathways including the phospholipase C (PLC)-γ, mitogen-activated protein kinase (MAPK), and phosphoinositide 3-kinase (PI3K)/AKT pathways, resulting in the differentiation, survival, and proliferation of cells [4, 5].

**Table 2:** Fusion genes seen in clinical studies of larotrectinib and entrectinib

Fusion	Number of patients	Number of patients with response	Response rate (%)
*NTRK1*	47	28	59.6
*CD74–NTRK1*	1	1	100.0
*CDC42BPA–NTRK1*	1	1	100.0
*CGN–NTRK1*	1	0	0.0
*CTRC–NTRK1*	1	0	0.0
*EPS15L1–NTRK1*	1	1	100.0
*ERC1–NTRK1*	1	0	0.0
*GON4L–NTRK1*	1	0	0.0
*IRF2BP2–NTRK1*	2	2	100.0
*LMNA–NTRK1*	7	3	42.9
*PDE4DIP–NTRK1*	1	1	100.0
*PDIA3–NTRK1*	1	0	0.0
*PEAR1–NTRK1*	2	0	0.0
*PLEKHA6–NTRK1*	2	1	50.0
*PPL–NTRK1*	1	1	100.0
*SQSTM1–NTRK1*	4	4	100.0
*TPM3–NTRK1*	13	7	53.8
*TPR–NTRK1*	5	5	100.0
*TRIM33–NTRK1*	1	0	0.0
*TRIM63–NTRK1*	1	1	100.0
*NTRK2*	2	1	50.0
*SQSTM1–NTRK2*	1	0	0.0
*STRN–NTRK2*	1	1	100.0
*NTRK3*	60	43	71.7
*AKAP13–NTRK3*	1	0	0.0
*EML4–NTRK3*	2	0	0.0
*ETV6–NTRK3*	50	38	76.0
*FAM19A2–NTRK3*	1	0	0.0
*Inferred ETV6–NTRK3*	3	3	100.0
*KIF7–NTRK3*	1	0	0.0
*RBPMS–NTRK3*	1	1	100.0
*TPM4–NTRK3*	1	1	100.0
Total	109	72	66.1

### NTRK gene alterations

Among various alterations of the *NTRK* genes, missense variants of the *NTRK* genes and *NTRK* fusion genes are important in terms of the treatment of malignant tumors.

#### Gene variants and amplification

The alteration of the *NTRK* genes has been reported in tumors such as colorectal cancer, lung cancer, malignant melanoma, and acute leukemia. However, TRK activity of these altered genes is similar to or lower than that of the wild type [6]. Although association between the missense variants of the *NTRK* genes and the development of malignant tumors has not been elucidated, it has been reported that if a tumor has the missense variants of the *NTRK* genes involving the kinase region, it becomes resistant to TRK inhibitors, larotrectinib, and entrectinib. Moreover, an *NTRK1* splice variant, TRKA III, and an in-frame deletion mutant (ΔTRKA) were reported in neuroblastoma and acute myeloid leukemia, showing their tumorigenicity [7, 8]. As for the association between the *NTRK* genes and diseases other than malignant tumors, congenital insensitivity to pain with anhidrosis type IV, a hereditary disease, has a pathological variant of the *NTRK1* gene. The amplification of the *NTRK* genes has been reported in tumors such as breast cancer, cutaneous basal cell cancer, and lung cancer. Although it has been reported that TRKA and TRKC expression in neuroblastoma indicates a good prognosis [9], its tumorigenicity or significance as a target of treatment has not yet been elucidated.

#### Fusion genes

*NTRK* fusion genes are tumorigenic genetic alterations reported in many cancer types [10]. Through intrachromosomal or interchromosomal translocation, a fusion gene is formed with a 3′ part of the *NTRK1–3* genes encoding the kinase region and a 5′ part of a partner gene (various genes have been reported). A ligand-independent kinase activation induced by the formation of a fusion gene is considered to contribute to carcinogenesis. Fusion genes seen in clinical studies of larotrectinib and entrectinib are shown in Table 2 (pooled results for 54 entrectinib patients and 55 larotrectinib patients based on the approval application materials).

### Frequency of *NTRK* fusion genes by cancer type

*NTRK* fusion genes are found in a wide variety of cancer types (Table 3) [5, 11–14]. *NTRK* fusion genes are frequently seen in some types of cancer. These include Secretory carcinoma of the salivary gland (mammary analog secretory carcinoma) [15, 16], Secretory carcinoma of the breast [17–19], infantile fibrosarcoma (congenital fibrosarcoma) [20–23], and congenital mesoblastic nephroma. The gene fusion seen in nearly all of these types of cancer is *ETS translocation variant 6* (*ETV6*)–*NTRK3* fusion. In other types of cancer, the frequency of the *NTRK* gene fusion is generally low (Table 3).

**Table 3:** Reported frequency of NTRK fusion in various types of tumors

Disease	Incidence by literature	Incidence by TCGA (*n* = 9,966) [5]
High frequency (> 50%)	Review article [11]	
Secretory carcinoma of the breast	92.87%	92%
Infantile fibrosarcoma	90.56%	86–91%
Secretory carcinoma of the salivary gland	79.68%	93–100%
Pigmented spindle cell nevus of Reed	56.52%	
Intermediate frequency (10–50%)	Review article [11]	
Papillary thyroid cancer	25.93%	
Differentiated thyroid cancer	22.22%	
congenital mesoblastic nephroma	21.52%	
Glioblastoma	21.21%	40%
Low-grade mucoepidermoid carcinoma	20.00%	
Acinar cell carcinoma of the salivary gland	11.11%	
Low frequency	FoundationCore (*n* = 217,086) [12]	
Salivary gland cancer	2.49%	
Thyroid cancer	1.07%	2.34%
Soft tissue sarcoma	1.06%	0.76%
GIST	0.59%	
Glioma	0.33%	0.56%
Peritoneal cancer	0.29%	
Fallopian tube cancer	0.28%	
Bladder cancer	0.23%	
Breast cancer	0.23%	0.18%
Colorectal caner	0.21%	0.97%
Liver cancer	0.19%	
Uterus cancer	0.19%	0.33% (cervical cancer)
Biliary cancer	0.18%	
Ovarian cancer	0.18%	
Non-small cell lung cancer	0.17%	0.18%
Bone sarcoma	0.16%	
Malignant melanoma	0.16%	0.21%
Bile duct cancer	0.15%	
Prostate cancer	0.15%	
Cancer of unknown primary	0.14%	
Gastric cancer	0.14%	
Pancreatic cancer	0.13%	0.56%
Small intestine cancer	0.10%	

As for secretory carcinoma of the salivary gland (mammary analog secretory carcinoma: MASC), Skalova et al. in the Czech Republic reported the presence of *ETV6–NTRK3* fusion genes in tumors that developed in the salivary gland histologically resembling secretory breast carcinoma in 2010 [24]. It has been reported that MASC is more frequently found in men, and the mean age of onset is 44 years [25].

Secretory breast carcinoma is a very rare breast cancer; its frequency is < 0.15% among all breast cancers, with the median age of onset of 25 years, and it is found in both males and females [26]. Secretory breast carcinoma is triple negative in many cases and has *ETV6–NTRK3* fusion genes. Although the prognosis is good, there have been reports of very late recurrence.

Infantile fibrosarcoma accounts for 12% of infantile malignant tumors. It has also been reported that 36–80% of infantile fibrosarcomas are congenital. It is rare that children 2 years of age or older develop infantile fibrosarcoma. Infantile fibrosarcoma frequently develops in limbs and has *ETV6–NTRK3* fusion genes. It has a better prognosis than adult fibrosarcoma. The efficacy of chemotherapy and cases of spontaneous regression have been reported [27]. Congenital mesoblastic nephroma [19] is the most frequent renal tumor in infants 3 months of age or younger. It is recognized as a low-grade tumor with a good prognosis. It infrequently develops in both kidneys and is sometimes accompanied by hypertension and hypercalcemia.

High-grade gliomas in children, particularly in infants younger than 3 years old, have better life prognoses than high-grade gliomas in older children and adults, and do not have alterations of the histone *H3.1* or *H3.3* gene, which are found in tumors in older children at a high frequency, or of the *isocitrate dehydrogenase* (*IDH*)* 1* or *IDH2* gene, which are found in tumors in young adults at a high frequency. Recently, it has been reported that *NTRK* fusion genes are found at a high frequency in infantile brain tumors in non-brain stem areas [28, 29].

As for lung cancer, in a study in 4872 patients at 7 institutions, *NTRK* fusion genes were found in 11 patients (0.23%). Of them, 6 patients (55%) were male, 8 patients (73%) were non-smokers/light smokers, and the median age was 47.6 years [30]. Nine of the 11 patients had adenocarcinoma. *NTRK* fusion genes were also detected in squamous cell carcinoma and neuroendocrine carcinoma.

In most gastrointestinal stromal tumors (GISTs), genetic alterations of *KIT* or *platelet-derived growth factor A* (*PDGFRA*) gene that activate their functions are detected, while wild-type GIST, in which these genetic alterations are not detected, accounts for approximately 10% of all GISTs. *NTRK* fusion genes are found in wild-type GISTs [31]. On the other hand, it has also recently been reported that gastrointestinal mesenchymal tumors with *NTRK* fusion genes are basically non-GIST, although this was found in a small study [32]. In the WHO Classification of Tumours, Soft Tissue and Bone Tumours, 5th Edition, the category "*NTRK*-rearranged spindle cell neoplasm (emerging)" was established for mesenchymal tumors in which *NTRK* fusion genes are seen [19].

### *NTRK* fusion gene testing

Methods for detecting *NTRK* fusion genes include testing by next-generation sequencing (NGS) methods, reverse transcription polymerase chain reaction (RT-PCR), fluorescence in situ hybridization (FISH), and immunohistochemistry (IHC) [33–36].

NGS testing includes not only DNA-based sequencing but also RNA-based sequencing, and each type has advantages and drawbacks. Comprehensive genome profiling tests that have received regulatory approval are OncoGuide™ NCC Oncopanel System [37] and FoundationOne® CDx Cancer Genome Profile [38]. In addition to these, tests such as the Oncomine™ Target Test, Todai OncoPanel, and TruSight Oncology 500 are being used in advanced medical care [39]. These tests examine gene alterations in tumor tissue. However, in March 2021, the FoundationOne^®^ Liquid CDx Cancer Genome Profile test, which detects gene alterations in the blood, received regulatory approval [40]. This made it possible to perform liquid biopsies, which offer advantages such as easy specimen collection and rapid results. However, a positive concordance rate of 47.4% in detecting *NTRK* fusion genes was found for the FoundationOne® Liquid CDx Cancer Genome Profile, for example [41]. Therefore, if the presence of an *NTRK* fusion gene is strongly suspected clinically and a liquid biopsy is negative for *NTRK* fusion genes, confirmation with another testing method should be considered. In DNA-based testing, the DNA is typically extracted from FFPE specimens, and detection is performed by amplicon sequencing or targeted hybridization capture. An advantage of NGS testing is that it can usually investigate not only *NTRK* fusion genes but also other gene alterations at the same time. For tests that are configured to detect only known fusion partners, it has been reported that false negatives are produced for unknown partners and that tiling of repeat regions and entire introns (e.g., the intron region of *NTRK3* is long, up to 193 KB), may decrease sensitivity for detecting chromosomal translocation and inversion. An advantage of RNA-based testing methods is that introns are spliced out. Some of them can detect *NTRK* fusion genes regardless of the fusion partner. Because RNA is more unstable than DNA, greater attention must be paid to specimen quality.

*NTRK* fusion genes have a broad variety of fusion partners and breakpoints. Consequently, reverse transcriptase polymerase chain reaction (RT-PCR) has limitations when used to examine *NTRK* fusion genes. In some types of cancer (e.g., mammary gland secretory carcinoma, salivary gland secretory carcinoma, and infantile fibrosarcoma), the fusion genes detected are limited to *ETV6*–*NTRK3* fusion genes in most cases. Although examination by RT-PCR is considered in such cases, if the presence of *NTRK* fusion genes is strongly suspected clinically and RT-PCR is negative for *NTRK* fusion, confirmation with another method should be considered. Recently, semi-specific RT-PCR has been used in efforts to detect fusion genes even when the fusion partner is unknown [42].

Fluorescence in situ hybridization (FISH) can easily determine the presence of fusion genes with any type of fusion gene partner, but it must be performed 3 times to investigate *NTRK1*–*3*. However, when *ETV6*–*NTRK3* fusion is expected (e.g., in mammary gland secretory carcinoma, salivary gland secretory carcinoma, and infantile fibrosarcoma), *NTRK3* alone needs to be examined and only a single test is required for the examination, the use of FISH is appropriate. FISH also has several limitations. In the case of intrachromosomal rearrangement (*LMNA*–*NTRK1* in particular), signal discrimination is difficult, which may lead to a false-negative result [43].

Immunohistochemistry (IHC) does not detect fusion genes directly but rather detects TRK protein expression. However, it is less expensive than other methods, and its use is under investigation. A study found that in IHC investigations using an antibody cocktail, false positives were common when there was no TRK protein expression, even though no *NTRK* fusion genes were seen [44]. Currently the most commonly used IHC test is clone EPR17341, a pan-TRK antibody (Abcam, Roche/Ventana). Cytoplasm stains positive in many cases, but staining of nuclei (ETV6, etc.) and cytomembrane (TPM, TPR, etc.) are also presented. Although no cutoff for positive results has been established, 1% or 10% is defined as positive in some reports. Depending on the report, sensitivity has ranged from 75% to 96.7% and specificity from 92 to 100% [33, 45–47]. With *NTRK3*, however, lower sensitivity has been reported, and caution is, therefore, required in this case [48]. If an *NTRK* fusion gene is strongly suspected clinically and TRK protein expression is negative by IHC, confirmation of the results by another method should be considered. In soft tissue sarcomas, brain tumors, and neuroblastomas, TRK expression is observed even if no *NTRK* fusion gene is seen, which has been noted to be prone to false positives [49].

There is also another method. A gene expression analysis developed by NanoString Technologies, Inc. uses probes with unique molecular fluorescent barcodes that are specific to the sequences of target molecules. The probes are hybridized with target nucleic acid and then fixed on the surface of a cartridge. The sequence of the color barcodes bound to each target sequence are digitally counted using a fluorescent scanner. This gene expression analysis is expected to obtain good counting results of RNA samples prepared from formalin-fixed paraffin-embedded (FFPE) specimens. Since there are no sufficient data regarding the detection of *NTRK* fusion genes, further studies are required in the future.

### TRK inhibitors

Those that have been approved in Japan are entrectinib and larotrectinib.

Entrectinib is an oral tyrosine kinase inhibitor that inhibits ROS1, TRK (and ALK). The results of a pooled analysis of the phase I studies ALKA-372-001 and STARTRK-1 and the phase II study STARTRK-2 [50] showed a response rate of 57.4% in 54 patients with cancers such as soft tissue sarcoma, non-small-cell lung cancer, and salivary gland secretory carcinoma [51]. Major adverse events included taste disorder (47.1%), constipation (27.9%), fatigue (27.9%), diarrhea (26.5%), peripheral edema (23.5%), dizziness (23.5%), and increased creatinine (17.6%) [51]. In addition, the STARTRK-NG study, which focused on children and adolescents, reported efficacy in cancers that included CNS tumors.

Entrectinib was designated a breakthrough therapy for *NTRK* fusion gene-positive solid tumors in May 2017 and approved in August 2019 by FDA. It was designated a PRIME (PRIority MEdicines) therapy by the EMA in October 2017 and approved in July 2020. In Japan, it was designated a product subject to the *Sakigake* designation system (scheme for rapid authorization) in March 2018 and received regulatory approval for the treatment of *NTRK* fusion gene-positive advanced and recurrent solid tumors on June 18, 2019.

Larotrectinib is an oral tyrosine kinase inhibitor that selectively inhibits TRK. The results of a pooled analysis of the phase I 20,288 study in adult patients with *NTRK* gene fusion and the phase I/II SCOUT study in children with the same condition, and phase II NAVIGATE study has been reported [52]. These studies included cancers such as salivary gland tumors, soft tissue sarcomas, and thyroid cancer, and 159 patients included in the pooled analysis showed a response rate of 79%. Major adverse events included fatigue, nausea, dizziness, vomiting, increased AST, and cough [53]. Larotrectinib was approved by the FDA on November 26, 2018, by the EMA in September 2019, and in Japan on March 23, 2021.

Although TRK inhibitors have shown efficacy in solid tumors with *NTRK* fusion genes and have been approved for such treatment, their effectiveness in other *NTRK* gene alterations (e.g., gene mutation and amplification) has not been established. Although there have been case reports indicating that larotrectinib was effective in patients with esophageal cancer with gene alterations including *NTRK* gene amplification in the absence of *NTRK* fusion genes [54], the extent to which TRK inhibitors exhibit efficacy against *NTRK* gene amplification has not been established. Therefore, their use other than the investigational use is currently not recommended.

Although the mechanism of resistance to TRK inhibitors such as entrectinib and larotrectinib has not been completely elucidated, it has been reported that the presence of certain *NTRK* gene alterations results in resistance to these TRK inhibitors. Typical examples are the mutations p.G667C and p.G595R in *NTRK1* and p.G623R, p.G696A, and p.F617L in *NTRK3* [55–57].

Next-generation TRK inhibitors are also being developed. For example, selitrectinib (LOXO-195, BAY2731954) is a selective TRK inhibitor that has been reported to be effective even in the presence of the above-mentioned *NTRK* gene mutations of the kinase domain. A clinical study of selitrectinib is currently under way [58]. Repotrectinib (TPX-0005) has been reported to be effective not only against *NTRK* gene alterations but also *ROS1* and *ALK* gene alterations and was granted breakthrough designation by the FDA [59].

### Clinical questions (CQs)

The following requirements have been prepared regarding the *NTRK* fusion testing performed to select patients who are likely to benefit from TRK inhibitors and the administration of them. The clinical recommendations propose the following 14 requirements in 3 CQs regarding the *NTRK* fusion testing performed to select patients who are likely to benefit from TRK inhibitors.

1. *NTRK* fusion gene testing is not recommended for patients with solid tumors that have genetic alterations mutually exclusive with *NTRK* fusion genes.
2. Testing that can detect *ETV6*–*NTRK3* fusion genes is strongly recommended for known cancer types in which *NTRK* fusion genes are detected at a high frequency.
3. *NTRK* fusion gene testing is recommended for all patients with metastatic or recurrent solid tumors other than those described above to determine whether TRK inhibitors are indicated.
4. *NTRK* fusion gene testing is recommended for patients with known cancer types in which *NTRK* fusion genes are detected at a high frequency even when their solid tumors are curable.
5. *NTRK* fusion gene testing should be considered for all patients with early solid tumors other than those described above to determine whether TRK inhibitors are indicated.
6. It is strongly recommended that *NTRK* fusion gene testing should be performed before the start of the standard treatment or during the standard treatment.
7. To determine whether TRK inhibitors are indicated, an NGS test whose analytical validity has been established is strongly recommended.
8. FISH is not recommended as a screening test for *NTRK* fusion genes.
9. RT-PCR is not recommended as a screening test for *NTRK* fusion genes.
10. Testing for *NTRK* fusion genes (particularly *ETV6–NTRK3* fusion genes) using FISH or RT-PCR may be performed for known cancer types in which *NTRK* fusion genes are detected at a high frequency.

If the result is negative, confirmation with a different test is recommended.

11. IHC should be considered as a screening test for *NTRK* fusion genes.
12. IHC is not recommended to determine whether TRK inhibitors are indicated.
13. The use of TRK inhibitors is strongly recommended.
14. The use of TRK inhibitors from the initial treatment is recommended.

Please keep in mind that these clinical recommendations will be revised in a timely manner, along with continuously and steadily advancing cancer treatment and new knowledge on biomarkers.

We will explain each CQ in detail.

## CQ1: targets of *NTRK* fusion gene testing

PubMed was searched using the following queries: "NTRK or neurotrophic tropomyosin receptor kinase," "neoplasm," and "tested or diagnos* or detect*." The same queries were used to search Cochrane Library. For the search period from January 1980 to August 2019, 70 articles were extracted from PubMed and 1 from Cochrane Library. In addition, 4 articles were retrieved by handsearching. In revising the guidelines, an additional literature search was performed for the period from September 2019 to January 2021 using the above-described queries, and 133 additional articles were extracted from PubMed and 1 from Cochrane Library. In the primary screening, 144 articles were extracted, and 77 were extracted in the secondary screening. A qualitative systematic review of these articles was then performed.

**Table:** 

**CQ1-1: Patients with locally advanced or metastatic solid tumors**
**Is *NTRK* fusion gene testing recommended for patients with metastatic/recurrent solid tumors?**
1. *NTRK* fusion gene testing is not recommended for patients with solid tumors that have genetic alterations mutually exclusive with *NTRK* fusion genes
Recommendation level: Not recommended [SR: 0, R: 0, ECO: 4, NR: 16]
2. Testing that can detect *ETV6*–*NTRK3* fusion genes is strongly recommended for known cancer types in which *NTRK* fusion genes are detected at a high frequency
Recommendation level: Strongly recommended [SR: 17, R: 3, ECO: 0, NR: 0]
3. *NTRK* fusion gene testing is recommended for all patients with metastatic or recurrent solid tumors other than those described above to determine whether TRK inhibitors are indicated
Recommendation level: Recommended [SR: 6, R: 14, ECO: 0, NR: 0]

Clinical studies of entrectinib and larotrectinib, TRK inhibitors, have been conducted in patients with unresectable or metastatic solid cancers irrespective of the line of treatment and have demonstrated high efficacy. *NTRK* fusion genes have been observed irrespective of cancer types, although at a low frequency. Moreover, no reliable indices that can determine the presence or absence of *NTRK* fusion genes in clinical settings have been established. Therefore, we strongly recommend the testing for all metastatic/recurrent solid cancers in which the presence of *NTRK* fusion genes has been reported, to determine whether TRK inhibitors are indicated [60]. We also strongly recommend the testing for tumors such as secretory carcinoma of the salivary gland (mammary analog secretory carcinoma), secretory breast carcinoma, infantile fibrosarcoma (congenital fibrosarcoma), congenital mesoblastic nephroma, and pediatric high-grade glioma (younger than 3 years old) because *ETV6–NTRK3* fusion genes are detected at a high frequency in these diseases (refer to "3. Frequency of NTRK fusion genes by cancer type"). Because *NTRK* fusion genes are mutually exclusive with other driver mutations, if mutually exclusive genetic alterations [e.g., *epidermal growth factor receptor* (*EGFR*) gene mutations, *anaplastic lymphoma kinase* (*ALK*) fusion genes, and *ROS1* fusion genes in non-small cell lung cancers; *rapidly accelerated fibrosarcoma* (*RAF*) gene mutations in malignant melanoma and colorectal cancer; and *KIT* gene mutations in GIST] of mitogenic pathways (groups of genes encoding the growth factor receptor, RAS, and MAPK pathways) are detected [48], a search for *NTRK* fusion genes is not necessary.

In conducting tests, aspects such as cost and frequency should also be considered and sufficiently discussed with the attending physicians and patients.

Information on approved in vitro diagnostics and medical devices for *NTRK* fusion gene testing is available at the following website:https://www.pmda.go.jp/review-services/drug-reviews/review-information/cd/0001.html

**Table:** 

**CQ1-2: Is *NTRK* fusion gene testing recommended for patients with early solid tumors?**
1. *NTRK* fusion gene testing is recommended for patients with known cancer types in which *NTRK* fusion genes are detected at a high frequency even when their solid tumors are curable
Recommendation level: Recommended [SR: 2, R: 12, ECO: 6, NR: 0]
2. *NTRK* fusion gene testing should be considered for all patients with early solid tumors other than those described above to determine whether TRK inhibitors are indicated
Recommendation level: Expert consensus opinion [SR: 0, R: 0, ECO: 19, NR: 1]

At present, the significance of TRK inhibitors as neoadjuvant/adjuvant therapy for patients with solid tumors possessing *NTRK* fusion genes has not been established. However, in a phase 1 study of larotrectinib in pediatric patients, a partial response was obtained following the administration of larotrectinib in 5 patients and resection was subsequently performed [61]. In 3 of them, tumors were completely resected. Because it has been reported that patients with metastatic or recurrent solid tumors possessing *NTRK* fusion genes had a high response rate to TRK inhibitors, *NTRK* fusion gene testing is recommended for patients with known cancer types in which *NTRK* fusion genes are detected at a high frequency (including those detected at a relatively high frequency in Table 3). *NTRK* fusion gene testing may also be considered for curable solid tumors other than the above-mentioned types, in view of conducting a neoadjuvant therapy. As is seen in the field of pediatrics in particular, the use of a TRK inhibitor is considered when a potentially curative treatment has not been designated as the standard treatment due to insufficient evidence or when the standard treatment is likely to lack efficacy. Therefore, *NTRK* fusion gene testing should be considered for this.

**Table:** 

**CQ1-3: When should *NTRK* fusion gene testing be performed?**
It is strongly recommended that *NTRK* fusion gene testing should be performed before the start of the standard treatment or during the standard treatment
Recommendation level: Strongly recommended [SR: 13, R: 5, ECO: 2, NR: 0]

At this point, there has been no study report that compared the effectiveness of the standard treatment and that of TRK inhibitors in patients with metastatic or recurrent solid tumors possessing *NTRK* fusion genes. A trial calculation found that examining a 30% improvement in PFS in a randomized controlled study would require a minimum study duration of 2696 months (*α = *0.05, *β = *0.2, 1:1 allocation) [62]. A controlled study is, therefore, infeasible. The efficacy of TRK inhibitors was shown in the first line, and a high response rate has been reported. To prevent the loss of therapeutic opportunity for a patient who should be treated with TRK inhibitors because of the progress of the disease, we strongly recommend that *NTRK* fusion gene testing should be performed before the start of the standard treatment or during the standard treatment.

## CQ2: Testing methods for detecting *NTRK* fusion genes

PubMed was searched using the following queries: “NTRK or neurotrophic tropomyosin receptor kinase,”” “neoplasm,” "neoplasm,” “NGS,” “In Situ Hybridization,” “IHX,” “NanoString,” and “Polymerase Chain Reaction.” The same queries were used to search Cochrane Library. For the search period from January 1980 to August 2019, 129 articles were extracted from PubMed and 5 from Cochrane Library. In addition, 1 article was retrieved by handsearching. In revising the guidelines, an additional literature search was performed for the period from September 2019 to January 2021 using the above-described queries, and 124 additional articles were extracted from PubMed and 1 from Cochrane Library. In the primary screening, 43 articles were extracted, and 34 were extracted in the secondary screening. A qualitative systematic review of these articles was then performed.

**Table:** 

**CQ2-1: Is an NGS test recommended to determine whether TRK inhibitors are indicated?**
To determine whether TRK inhibitors are indicated, an NGS test whose analytical validity has been established is strongly recommended
Recommendation level: Strongly recommended [SR: 19, R: 1, ECO: 0, NR: 0]

In the development of entrectinib and larotrectinib, a variety of testing methods, such as NGS, FISH, and RT-PCR, have been used to determine whether TRK inhibitors are indicated. Because reported *NTRK* fusion genes vary over *NTRK1–3* genes and have various fusion partners, an NGS that can detect fusion genes of all *NTRK1–3* genes is recommended. A study in 33,397 patients that used an RNA-based panel test (MSK-Fusion) as a control reported sensitivity of 81.1% and specificity of 99.9% with a DNA-based panel sequence and sensitivity of 87.9% and specificity of 81.1% with IHC (clone EPR17341) [48]. Sensitivity and specificity were poor in sarcomas in this study, and the RNA-based panel test was recommended. Although liquid biopsies have also been approved, the positive predictive value of some of the biopsies for *NTRK* fusion genes is not necessarily high. Therefore, to what extent the gene panel and the type of specimen allow for detecting *NTRK* fusion genes should be determined. NGS tests include tests that can detect only known fusion partners and those that are capable of detection regardless of the fusion partner. Tests whose analytical validity has been established (e.g., approved in vitro diagnostics or medical devices) are recommended. Although the use of FFPE specimens is assumed in routine clinical practice, compliance with separately established guidelines (Guidelines on the Handling of Pathological Tissue Samples for Genomic Research/Treatment, ed., Japanese Society of Pathology) is recommended for the process from the fixation and storage of specimens to the extraction of the DNA and RNA.

To detect *NTRK* fusion genes, FoundationOne^®^ CDx Cancer Genome Profile and FoundationOne^®^ Liquid CDx Cancer Genome Profile have been approved as companion diagnostics for entrectinib, and FoundationOne^®^ CDx Cancer Genome Profile has been approved as a companion diagnostic for larotrectinib. Although these tests can detect *NTRK1*, *NTRK2*, and *NTRK3* fusion genes, it should be noted that they do not detect *NTRK3* intron regions.

To detect *NTRK* fusion genes, a test whose analytical validity has been established is recommended, both when used as companion diagnostics and as part of comprehensive genome profile testing. In the latter case, genes other than *NTRK* fusion genes are also investigated. Therefore, when cancer genome profiling tests are performed, the Guidelines for Establishing Core Hospitals, etc. for Cancer Genomic Medicine (partially revised on July 19, 2019) and the guidelines of the relevant academic societies should first be referred to.

**Table:** 

**CQ2-2: Are FISH and RT-PCR recommended for the detection of *NTRK* fusion genes?**
1. FISH is not recommended as a screening test for *NTRK* fusion genes
Recommendation level: Not recommended [SR: 0, R: 0, ECO: 2, NR: 18]
2. RT-PCR is not recommended as a screening test for *NTRK* fusion genes
Recommendation level: Not recommended [SR: 0, R: 1, ECO: 5, NR: 14]
3. Testing for *NTRK* fusion genes (particularly *ETV6-NTRK3* fusion genes) using FISH or RT-PCR may be performed for known cancer types in which *NTRK* fusion genes are detected at a high frequency
Recommendation level: Expert consensus opinion [SR: 0, R: 8, ECO: 12, NR: 0]

If the result is negative, confirmation with a different test is recommended.

Recommendation level: Recommended [SR: 7, R: 8, ECO: 5, NR: 0].

Because *NTRK* fusion genes vary over *NTRK1–3*, FISH and PCR have limitations in detecting them. For FISH, break-apart probes for *NTRK1–3* have been reported to be used, and 3 FISH assays are required in screening. It should be noted that with intrachromosomal rearrangements, which are seen with alterations such as *NTRK1* fusion genes, false negatives results may occur. Regarding PCR, because the preservation of RNA in FFPE is problematic and the ranges of partner genes are unknown, it is not possible to judge what degree of detection accuracy can be ensured for PCR. Therefore, PCR cannot be recommended. However, if single gene tests that can solve these problems are developed, the PCR method needs to be reexamined. Although amplicon sequencing is based on the same principle as the PCR method, it can detect other genetic alterations and the detection accuracy has been specified. Therefore, amplicon sequencing will be discussed along with NGS.

In cancers such as salivary gland secretory carcinoma (mammary analog secretory carcinoma), mammary gland secretory carcinoma, infantile fibrosarcoma (congenital fibrosarcoma), and congenital mesoblastic nephroma, testing with FISH and PCR may be considered because nearly all the fusion genes seen are *ETV6*-*NTRK3*. If the result is negative, however, confirmation with a different test is recommended.

Finally, a separate report indicated that there are cases in which fusion genes cannot be detected with IHC, FISH, or NGS [63]. Caution must, therefore, be exercised regarding findings such as false-positive and false-negative results of individual tests, and close collaboration between the clinician and diagnostic pathologist is important [64]. In particular, if *NTRK* fusion genes are not detected in known cancer types in which *NTRK* fusion genes are detected at a high frequency, it is desirable to confirm the results by other testing methods.

**Table:** 

**CQ2-3: Is IHC recommended to detect *NTRK* fusion genes?**
1. IHC should be considered as a screening test for *NTRK* fusion genes
Recommendation level: Expert consensus opinion [SR: 0, R: 11, ECO: 8, NR: 1]
2. IHC is not recommended to determine whether TRK inhibitors are indicated
Recommendation level: Not recommended [SR: 0, R: 0, ECO: 0, NR: 20]

IHC is a method of detecting TRK proteins. Because even a positive IHC result does not indicate the presence of an *NTRK* fusion gene, IHC is not recommended as a test to determine whether a TRK inhibitor is indicated. However, there has been a report of a study using an antibody cocktail, in which *NTRK* fusion genes were not detected when IHC was negative. Therefore, NGS or other tests can be omitted when IHC was negative, and IHC is expected to be valid as a screening test. An assay widely used in investigations is clone EPR17341 (Abcam, Roche/Ventana), a pan-TRK antibody. Its sensitivity has been reported to be 75–96.7% and its specificity 92–100%. However, caution is needed for *NTRK3* due to lower sensitivity. The results of IHC tests should be interpreted carefully because sensitivity and specificity differ depending on the antibody used; because false positives have been reported in cancers such as soft tissue sarcomas, brain tumors, and neuroblastomas due to TRK protein expression in these cancers; and because evaluation criteria have not been adequately established. However, because test results can be obtained rapidly and the tests are inexpensive, their further development is expected in the future.

## CQ3: treatment for *NTRK* fusion genes

PubMed was searched using the following queries: "NTRK or neurotrophic tropomyosin receptor kinase," "neoplasm," "treatment," and "TRK inhibitor." The same queries were used to search Cochrane Library. For the search period from January 1980 to August 2019, 132 articles were extracted from PubMed and 6 from Cochrane Library. In addition, 2 articles were retrieved by handsearching. In revising the guidelines, an additional literature search was performed for the period from September 2019 to January 2021 using the above-described queries, and 180 additional articles were extracted from PubMed and 1 from Cochrane Library. In the primary screening, 88 articles were extracted, and 43 were extracted in the secondary screening. A qualitative systematic review of these articles was then performed.

**Table:** 

**CQ3-1: Are TRK inhibitors recommended for unresectable/metastatic/recurrent solid cancers possessing *NTRK* fusion genes?**
The use of TRK inhibitors is strongly recommended
Recommendation level: Strongly recommended [SR: 20, R: 0, ECO: 0, NR: 0]

The efficacy of entrectinib and larotrectinib, TRK inhibitors, for solid tumors possessing *NTRK* fusion genes has been demonstrated. There have been no controlled studies that have compared TRK inhibitors with other drugs. However, a trial calculation found that examining a 30% improvement in PFS in a randomized controlled study would require a minimum study duration of 2696 months (*α = *0.05, *β = *0.2, 1:1 allocation) [61]. A controlled study is, therefore, infeasible. Response rates with TRK inhibitors are high, and adverse events are mild. Consequently, the benefits of TRK inhibitors are considered to greatly outweigh the negative effects. It is also unlikely that the preference of patients varies. From these considerations, the use of TRK inhibitors is strongly recommended for solid cancers possessing *NTRK* fusion genes.

If the standard treatment is available for the target cancer type, whether a patient should be treated with the standard treatment or TRK inhibitors should be determined individually, taking into consideration anticipated effects, expected adverse events, and late toxicity of respective treatments.

**Table:** 

**CQ3-2: When should TRK inhibitors be used?**
The use of TRK inhibitors from the initial treatment is recommended
Recommendation level: Recommended [SR: 7, R: 11, ECO: 2, NR: 0]

The efficacy of entrectinib is seen beginning from the initial treatment. Although there have been no controlled studies that have directly compared TRK inhibitors with other drugs, response rates with TRK inhibitors are high, and adverse events are mild. Thus, the benefits of TRK inhibitors are considered to greatly outweigh the negative effects. The use of an TRK inhibitor is, therefore, recommended beginning from the initial treatment. The same recommendation also applies to rare diseases with no standard treatment.

If the standard treatment is available for the target cancer type, whether a patient should be treated with the standard treatment or TRK inhibitors should be determined individually, taking into consideration the patient's background, anticipated effects, expected adverse events, and late toxicity of respective treatments. Because the long-term effects of TRK inhibitors in infantile fibrosarcoma have not been determined, no consensus on their use in the initial treatment of this condition has been established [65].

## Conclusion

*NTRK* fusion is a rare but significant target for treatment across tumor types. Clinicians must properly identify such rare but critical therapeutic targets to avoid missing the chance to provide therapeutic agents at the right time, through the right way, and to the right patients. In this guideline, the panel recommends the requirements for performing *NTRK* testing properly to select patients who are likely to benefit from TRK inhibitors.
